# Echinocandins: structural diversity, biosynthesis, and development of antimycotics

**DOI:** 10.1007/s00253-020-11022-y

**Published:** 2020-12-03

**Authors:** Wolfgang Hüttel

**Affiliations:** Institute of Pharmaceutical Sciences, University of Freiburg, Albertstr. 25, 79106 Freiburg, Germany

**Keywords:** Bioactivity, Filamentous fungi, Hydroxylases, Metabolic diversity, Non-ribosomal peptide biosynthesis, Secondary metabolism

## Abstract

**Abstract:**

Echinocandins are a clinically important class of non-ribosomal antifungal lipopeptides produced by filamentous fungi. Due to their complex structure, which is characterized by numerous hydroxylated non-proteinogenic amino acids, echinocandin antifungal agents are manufactured semisynthetically. The development of optimized echinocandin structures is therefore closely connected to their biosynthesis. Enormous efforts in industrial research and development including fermentation, classical mutagenesis, isotope labeling, and chemical synthesis eventually led to the development of the active ingredients caspofungin, micafungin, and anidulafungin, which are now used as first-line treatments against invasive mycosis. In the last years, echinocandin biosynthetic gene clusters have been identified, which allowed for the elucidation but also engineering of echinocandin biosynthesis on the molecular level. After a short description of the history of echinocandin research, this review provides an overview of the current knowledge of echinocandin biosynthesis with a special focus of the diverse structural elements, their biosynthetic background, and structure−activity relationships.

**Key points:**

• *Complex and highly oxidized lipopeptides produced by fungi.*

• *Crucial in the design of drugs: side chain, solubility, and hydrolytic stability.*

• *Genetic methods for engineering biosynthesis have recently become available.*

**Supplementary Information:**

The online version contains supplementary material available at 10.1007/s00253-020-11022-y.

## Introduction

Echinocandins are cyclic non-ribosomal hexapeptides equipped with a lipophilic side chain. They are produced by filamentous fungi (Ascomycota) of the classes Leotiomycetes (mostly Helotiales) and Eurotiomycetes (Aspergillaceae*)*. Thus, echinocandin biosynthesis is widespread albeit not frequent among Ascomycetes (Yue et al. 2015). Due to their strong inhibitory effect on 1,3-β-D-glucan synthase, an enzyme required for cell wall biosynthesis in fungi, echinocandins are potent antifungal compounds. The semisynthetic derivatives caspofungin (Cancidas*®*), micafungin (*Mycamine®*), and anidulafungin (Eraxis®) are first-line antimycotics for the treatment of invasive mycosis (Denning 2002; Patil and Majumdar 2017). Echinocandins have a distinctive cyclic lipopeptide structure assembled by a non-ribosomal peptide synthase (NRPS) (cf. Fig. 2). Six amino acids, up to five of which are non-proteinogenic, form a strictly conserved hexapeptide backbone (Hüttel 2017). In contrast, variations at the side chains are frequent, most of them due to exchange by similar amino acids or incomplete hydroxylation by one of the diverse hydroxylases. While the majority of the functional groups in the side chains are actually not essential for bioactivity (Zambias et al. 1992), they may enhance or reduce bioactivity, solubility, and other pharmacological properties. The peptide ring is closed via an unusual *N*-acyl-hemiacetal bridge between dihydroxy-l-ornithine (amino acid position **1**) and hydroxy-l-proline (position **6**). It is comparatively stable to hydrolysis; nevertheless, the group is critical for the stability of echinocandins. The fatty acid side chain originates either from primary metabolism, or, in the case of branched chains, it is biosynthesized by a polyketide synthase (PKS).

## Historical background of echinocandin research and development

The echinocandins are an outstanding example of basic and applied research by pharmaceutical industry. Only since the genomic age, almost four decades after the discovery of echinocandin B, has academic research dominated due to the elucidation of the molecular genetic basis of echinocandin biosynthesis. Echinocandin B was first discovered in 1974 by a group from Ciba-Geigy AG (Switzerland) as a metabolite of *Aspergillus delacroxii* (formerly *Aspergillus nidulans* var. *echinolatus* A 32204) (Benz et al. 1974). Independently, researchers from Sandoz AG (Switzerland) reported echinocandin B from *Aspergillus rugulosus* strain NRRL 8039 (Keller-Juslén et al. 1976), and Ely Lilly & Co. (USA) patented echinocandin B and derivatives as antimycotic factors A-30912 A–F produced by *A. rugulosus* strain NRRL 8113 (Hoehn and Michel 1977). At about the same time, a related group of echinocandins, the aculeacins, were isolated from *Aspergillus aculeatus* M 4214 by the Research Laboratories of Toyo Jozo Co. (Japan) (Mizuno et al. 1977; Satoi et al. 1977). Later, in 1989, Merck & Co. Research Laboratories (USA) reported pneumocandin A_0_ as a metabolite of the Helotiales species *Glarea lozoyensis* ATCC 20868 (formerly *Zalerion arboricola*) (Fromtling and Abruzzo 1989; Schwartz et al. 1989; Wichmann et al. 1989). Research and development on pneumocandin A_0_ and derivatives was intensively continued and, about three years later, in 1992, no less than 14 scientific articles regarding production, metabolic spectrum, biosynthesis, structure−activity relations, and chemical modification of pneumocandins had been published (Adefarati et al. 1991; Adefarati et al. 1992; Balkovec and Black 1992; Balkovec et al. 1992; Bartizal et al. 1993; Hammond 1993; Hensens et al. 1992; Masurekar et al. 1992; Mukhopadhyay et al. 1992; Schmatz et al. 1992; Schwartz et al. 1993; Schwartz et al. 1992; Tkacz et al. 1993; Zambias et al. 1992). For a detailed overview on the echinocandins and their producer strains, see Tables S3 and S4, respectively, and previous reviews (Balkovec 1994; Hino et al. 2001); Hüttel (2017); (Mukhopadhyay et al. 1999). Currently, strains of more than 20 fungal species are reported to produce echinocandins, of which echinocandin B from diverse *Aspergilli* (*Emericella*) is the most common product (Table S4).

**Fig. 1:**
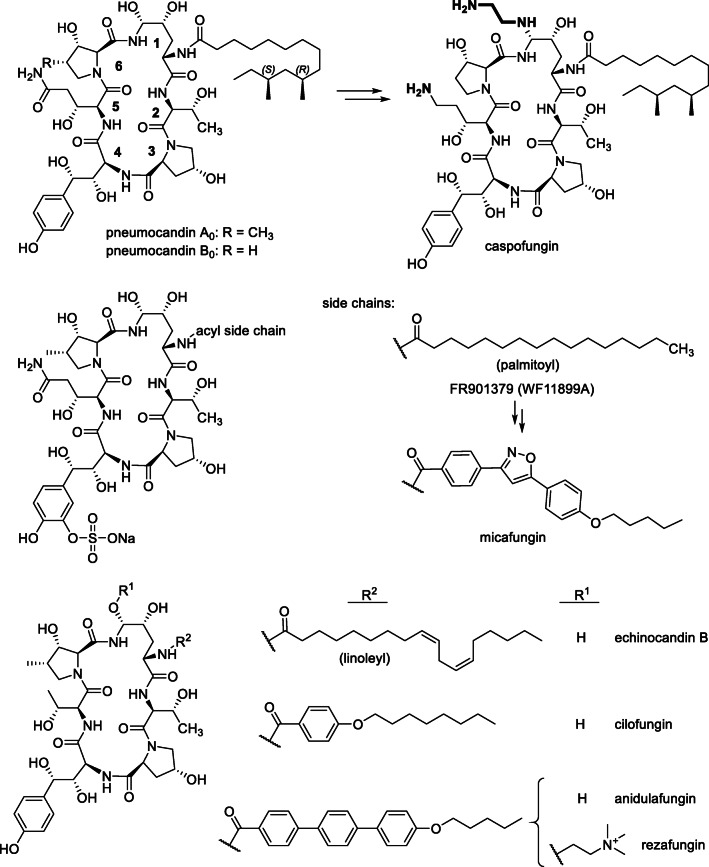
Structures of echinocandin natural products (pneumocandin A_0_ and B_0_, FR901379, and echinocandin B) and, derived therefrom, semisynthetic drugs (caspofungin, micafungin, and anidulafungin) or drug candidates (cilofungin and rezafungin)

Research and development of echinocandins was from the beginning focused on their application as antimycotics. Due to the complex structure, chemical total synthesis, even of simplified structures with non-hydroxylated side chains (i.e., the use of proteinogenic amino acids) was economically not feasible (Kurokawa and Ohfune 1986a; Kurokawa and Ohfune 1986b; Messik and Oberthür 2013; Zambias et al. 1992), so that fermentation in combination with chemical modification was the only economical option. The relevant fungal strains were well cultivatable and produced echinocandins in reasonable amounts; however, considerable development efforts were necessary to produce echinocandins effectively in industrial scale. These included the development of optimized fermentation processes, modification of the product spectrum through mutagenesis, and chemical modification towards compounds with improved efficacy profile and better solubility. On a structural level, the linear fatty acid side chain was replaced in anidulafungin and micafungin semisynthesis to avoid hemolytic side effects, and amino groups were introduced in caspofungin semisynthesis to increase activity and solubility. The first echinocandin drug candidate was cilofugin developed by Eli Lilly & Co. (USA), an echinocandin B derivative with a 4-octyloxy-benzoate side chain (Fig. 1) reducing hemolytic side effects (reviews, Balkovec et al. 2014; Connors and Pollard 2004; Debono and Gordee 1994; Li et al. 2018; Schwartz et al. 1993). However, as cilofungin was hardly water-soluble, polyethylene glycol was used as cosolvent to administer the drug which proved to be toxic (Kurtz and Rex 2001). Despite of this failure, Merck & Co. (USA) started their massive research and development initiative on pneumocandins in the 1990s, which finally led to the approval of caspofungin acetate (Candidas®) in 2001 (reviews, Balkovec et al. 2014; Connors and Pollard 2004; Debono and Gordee 1994; Kurtz and Rex 2001; Li et al. 2018; Schwartz et al. 1993). Caspofungin resulted from two structural modifications in pneumocandin B_0_: the interconversion of the hemiaminal into an *N*-ethyl aminal and the reduction of the hydroxyglutamine (position **5**) terminus to the amine, which effected a substantial improvement of solubility, stability, and pharmacological activity, especially also against *Aspergillus* species. For production, a mutant strain producing pneumocandin B_0_ instead of pneumocandin A_0_ was designed. Through fermentation, optimization and mutagenesis pneumocandin production could be increased from 5.8 mg L^−1^ to more than 1.2 g L^−1^ pneumocandin A_0_ (Masurekar et al. 1992*;* Schwartz et al. 1989). More recent approaches with *G. lozoyensis* mutant strains yielded pneumocandin B_0_ titers of up to 2.7 g L^−1^ (Qin et al. 2016; Song et al. 2018a, b, c). This corresponds to an overall increase of pneumocandin B_0_ productivity by a factor of > 3.500. For echinocandin D, more than 2.4 g L^−1^ have been reported recently with an *Aspergillus nidulans* mutant strain (Hu et al. 2020). The production of antibiotic FR901379 by *Coleophoma cylindospora* (*C. empetri*) could be increased by a factor of 30 (Kanda et al. 2009). After the successful development of caspofungin, two additional echinocandin antimycotics were launched. Micafungin (Mycamine®, Fujisawa Healthcare Inc., now Astellas Pharma Inc), a side chain derivative of FR901379 (Fig. 1), was approved in 2005 (Fujie 2007; Hashimoto 2009). A characteristic feature is a sulfate group at the dihydroxyhomotyrosine (position **4**) side chain that guarantees a good solubility. Finally, in 2006, anidulafungin was approved (Eraxis®, Vicuron Pharmaceuticals Inc., now Pfizer Inc.), which may be regarded as a cilofungin derivative with an improved side chain (Fig. 1) (Norris et al. 2008)

.

Due to their high and specific efficacy, the relatively good tolerability and the comparatively low development of resistance (although increasing in the last years), the introduction of echinocandins meant a considerable expansion of the possibilities for the treatment of invasive mycoses (reviewed by, e.g., (Aruanno et al. 2019; Mroczynska and Brillowska-Dabrowska (2020); Patil and Majumdar 2017). Currently, rezafungin developed by Cidara Therapeutics (USA) is in clinical phase III trial. The compound, an anidulafungin in which the hemiaminal bridge is substituted by a hydrolytically much more stable trimethylammonio acetal (Fig. 1), must be administered only once a week (Krishnan et al. 2017; Sofjan et al. 2018; Zhao et al. 2016).

## Biosynthesis

**Fig. 2:**
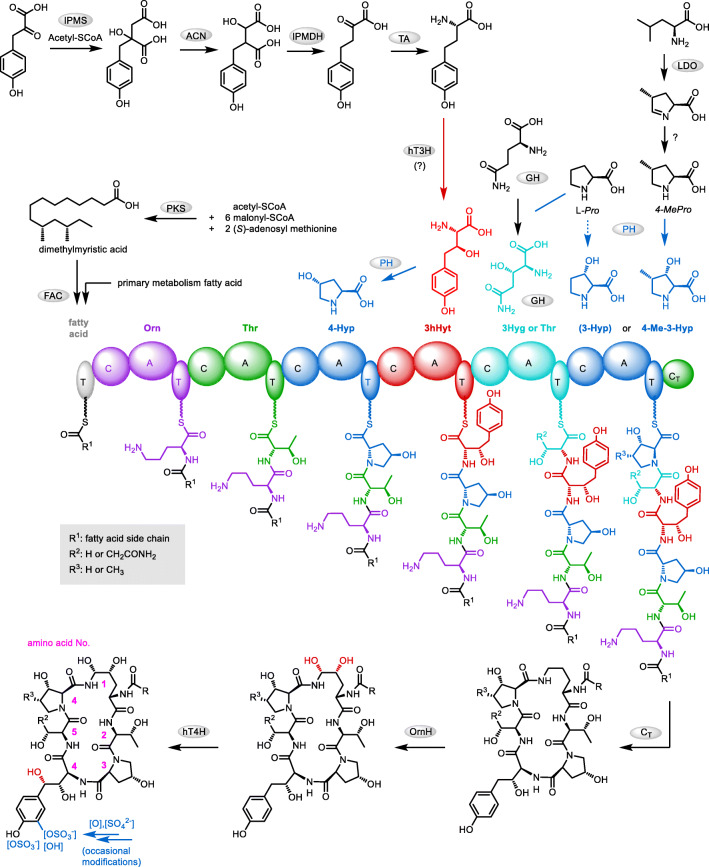
Overview of echinocandin biosynthesis. (A) Enzymes: FAC, long-chain fatty acyl-CoA ligase; IPMS, isopropylmalate synthase; ACN, aconitase; IPMDH, isopropylmalate dehydrogenase; TA, transaminase; LDO, l-leucine dioxygenase; PH, l-proline hydroxylase; hT3H, l-homotyrosine 3-hydroxylase; GH, putative l-glutamine hydroxylase; OrnH, l-ornithine 4,5-hydroxylase; hT4H, putative l-homotyrosine 4-hydroxylase; PKS, polyketide synthase (see also Table S2). Non-proteinogenic amino acids (all in l-configuration): 3Hyp, *trans*-3-hydroxyproline; 4Hyp, *trans*-4-hydroxyproline; 3hHyt, (3*S*)-3-hydroxyhomotyrosine; 3Hyg, (3*S*)-3-hydroxyglutamine; 4Me3Hyp, (3*S*,4*S*)-3-hydroxy-4-methylproline

Echinocandin biosynthesis was originally elucidated by researchers from Merck & Co. by means of ^13^C-labeling experiments with the pneumocandin producer *G. lozoyensis* (Adefarati et al. 1991; Adefarati et al. 1992). About 20 years later, the groups of Tang and Walsh made a breakthrough at the genetic level with the discovery and characterization of the echinocandin B biosynthetic gene cluster in *Aspergillus pachycristatus* NRRL 11440 (formerly: *Emericella rugulosa*) (Cacho et al. 2012; Jiang et al. 2013). This allowed for the first time a targeted gene deletion resulting in mutants producing novel echinocandin derivatives and heterologous expression of biosynthetic enzymes. The gene cluster has originally been described as being divided into two sections, *ecd* and *hty*, located on distant parts of the genome; however, it has been shown later by sequence comparison and PCR experiments that it is in fact a coherent biosynthetic gene cluster (*Ecd/hty*) (Hüttel et al. 2016). Heterologous expression and gene deletions, in particular of some of the oxygenases, already allow for a relatively clear picture of echinocandin biosynthesis as shown in Fig. 2 (more details in “Structural elements” section). To date, ten biosynthetic gene clusters are known, five from Leotiomycetes (*Ce_1*–*3*, *Gl/Glo*, and *PH*) and five from Eurotiomycetes (*Ecd/hty*, *Ani*, *AE*, *AA*, *and PA*) (Fig. S1, Table S1) (Hüttel 2017; Lan et al. 2020; Li et al. 2018). Phylogenetic analyses revealed that echinocandin biosynthesis has evolved in most cases from a common ancestor into strictly monophyletic clades (Yue et al. 2015). Since the two fungal classes diverged about 290 to 390 million years ago, echinocandin biosynthesis must have evolved in an ancestor species before that time. Considering this long timespan, the enzymes share remarkable high sequence identities of typically 50–70%, even between the two classes. More recently, it has been shown that acrophiarin biosynthesis in *Penicillium arenicola* NRRL 8095 (proposed genus *Phialomyces*) is structurally and evolutionarily a hybrid of Leotiomycetes and Eurotiomycetes (Helotiales) echinocandin biosynthesis (Lan et al. 2020). This is best explained by a horizontal gene transfer between a *P. arenicola* ancestor (Helotiales) and a Leotiomycetes strain, both possessing an echinocandin biosynthetic gene cluster. In addition, a phylogenetic relationship between the echinocandin and flutamide lipopeptide biosynthesis has been found (Yeh et al. 2016)

.

## Structural elements

The chemical and pharmacological properties of echinocandins are determined by the combination and interplay of all structural elements. However, in order to understand the system and to develop optimized compounds, it is important to first know the structure–activity relationships and biosynthesis of the individual building blocks. These will be discussed briefly in the following, even though the available information is often complex. As indicated above, all echinocandins share a consensus amino acid sequence plus one fatty acid (Table 1, cf. Fig. 2). Deviations in the side chain decoration, however, are frequent. It is remarkable that most congeners are also bioactive (Table S3). Therefore, the increasing resistance to echinocandins can possibly be countered with a variety of different structures. It is not unlikely that the intrinsic diversity in the biosynthesis of, e.g., pneumocandins is also directed against resistances (Hüttel 2017). In this section, the six amino acids and the fatty acid are discussed individually in terms of structural variations, their importance for bioactivity, and biosynthesis. As the bioactivity data are complex and depend much on target organisms and testing conditions, they are described here only in general terms such as “active,” “significantly reduced activity,” or “largely inactive.” For the more bioactive structures, minimum inhibitory concentrations (MIC) against echinocandin sensitive *Candida* and *Aspergillus* strains are usually in the sub-micromolar range (Klein et al. 2000; Patil and Majumdar 2017). An overall scheme of echinocandin biosynthesis, which may be referred to in the following discussion, is shown in Fig. 2. A more detailed overview on the individual (biosynthetic) echinocandin structures, their producer strains, and biosynthetic gene clusters can be found in the Supporting information.

**Table 1:** Prevalent amino acids in echinocandins

Position	Amino acid	Remark
**1**	4,5-l-Dihydroxyornithine	α-Acylated with a fatty acid (dimethyl myristate or from primary metabolism) δ-acylated with proline-**6**
**2**	l-Threonine (l-serine)	
**3**	*trans*-4-Hydroxyl-l-proline (4Hyp)	
**4**	Dihydroxy-l-homotyrosine	
**5**	3-Hydroxy-l-glutamine or l-threonine (l-serine)	
**6**	3-Hydroxy-4-methyl-l-proline (4Me3Hyp)	Connected with **1** via *C*-terminus

All echinocandins are acylated with a fatty acid attached to the α-amino group of dihydroxyornithine (position **1**). It is required for anchoring in the cell membrane and is thus essential for bioactivity (Boeck et al. 1989; Taft and Selitrennikoff 1990). Most producer strains recruit the fatty acid from primary metabolism, such as palmitoic, lineloic, or myristoic acid. However, species from Leotiomycetes incorporate a branched chain fatty acid, typically (10*R*,12*S*)-10,12-dimethylmyristate. This is synthesized by a highly reducing PKS with a methyl transferase domain, whose gene is included in the biosynthetic gene cluster (Fig. S1). From a pharmacological point of view, this is a clear advantage. In contrast to echinocandins with a linear fatty acid chain, (most) pneumocandins with a myristate-branched side chain have no considerable hemolytic side effects (Debono et al. 1988; Hashimoto 2009; Schmatz et al. 1992). Consequently, the original fatty acid is replaced in the antimycotic agents cilofungin, anidulafungin, and micafungin but not in caspofungin (Fig. 1). For this modification, Eli Lilly & Co. developed an in vivo process, in which echinocandin B is selectively deacylated by *Actinoplanes utahensis* in 60–70% yield (Boeck et al. 1989; Debono et al. 1989). The echinocandin B “nucleus” is then chemically re-acylated to cilofungin and other echinocandin B congeners using an active ester of the corresponding acid. Micafungin is produced from antibiotic FR901379 according to the same principle; however, *A. utahensis* has been replaced by a more efficient acylase from *Streptomyces* sp. No. 6907 (Fujie 2007). More recently, the acylation with a fatty acid has been investigated using genetic methods (Chen et al. 2016). Upon deletion of the fatty acid ligase gene in *G. lozoyensis*, none of the expectable pneumocandin “nucleus” structures were discovered, so the enzyme is most likely essential for echinocandin biosynthesis. However, a mutant lacking the PKS responsible for dimethyl myristate biosynthesis was able to partially compensate this deficiency with linear C14–C16 fatty acids, which shows that the ligase is not strictly substrate-specific. The effect was strongly enhanced by feeding the corresponding fatty acids.

The “default” amino acid **1** is (4*R*,5*R*)-4,5-dihydroxyornithine (dhOrn). In addition, there are a number of minor products in which ornithine is singly or not at all hydroxylated (Table S3). As indicated before, the *N*-acyl-hemiaminal bridge from the ornithine terminus to hydroxyproline in position **6** is sensitive to hydrolysis. Due to the reduced basicity of amide nitrogen and conformational constraints, the hemiaminal is reasonably stable at weakly acidic to neutral conditions and begins to hydrolyze at pH > 7 (Balkovec et al. 1992; Schwartz et al. 1992). The ornithine aldehyde resulting from hydrolysis forms an even more stable 5-membered cyclic hemiaminal with the acylated α-amino group of ornithine. The hydroxyl groups of dihydroxyornithine are apparently not essential for bioactivity. Ornithine derivatives of echinocandins, e.g., pneumocandin A_2_ and B_2_, echinocandin D, and various synthetic echinocandins, were found to be highly bioactive (Klein et al. 2000; Schmatz et al. 1992; Zambias et al. 1992). For the biosynthesis of the *N*-acyl-hemiaminal bridge to hydroxyproline **6**, the chemical reactivity demands that the hydroxylation at C-5 must occur after cyclization of the peptide via the δ-amino group. Otherwise, in the case of δ-hydroxylation of free ornithine, the resulting hemiaminal would rapidly hydrolyze ruling out the formation of the bridge. Accordingly, a cytochrome-P450 (CYP)-dependent hydroxylase (OrnH, Fig. 2) was found in *A. pachycristatus* NRRL 11440, which introduces both hydroxyl groups into the corresponding cyclic precursor of echinocandin B (Jiang et al. 2013).

The amino acid at position **2** in echinocandins is usually ʟ-threonine. Exceptions are sporiofungin A and C from *Pezicula radicolata* and Fr209602–4 from *Coleophoma cylindospora*, in which serine is found. ʟ-serine derivatives have also been found in minor fractions of pneumocandin production by *G. lozoyensis* (Table S3). Serine incorporation could be enhanced by feeding the amino acid (Connors and Pollard 2004). Little is known on the structure–function relationships regarding these amino acids.

Positions **3** and **6** are occupied by different hydroxy-l-prolines (Hyp). While position **3** is *trans*-4-hydroxy-l-proline (4Hyp) in all naturally occurring echinocandins, (2*S*,3*S*,4*S*)-3-hydroxy-4-methyl-l-proline (4Me3Hyp) is the dominating amino acid at position **6**. In some biosynthetic minor products, 4Me3Hyp is replaced by *trans*-3-Hyp (3Hyp); the most prominent example is pneumocandin B_0_, the precursor of caspofungin, produced by *G. lozoyensis* (Table S3). This fungus generally shows an exceptionally promiscuous incorporation of l-proline derivatives, which led to a pneumocandin notification as A–E based on proline **6** (pneumocandin A, 4Me3Hyp; B, 3Hyp; C, 4Hyp; D, (2,3-*trans*, 2,4-*trans*)-3,4-dihydroxy-l-proline; E, l-proline; see also Table S3).

As for the other amino acids in echinocandins, no specific substitution pattern is necessary on the prolines to maintain bioactivity. The bioactivity data are complex: pneumocandins A–D and synthetic l-proline derivatives of cilofungin (cf. Fig. 1, positions **6** and **3**) were found to be active against fungal pathogens in vitro (Balkovec et al. 1992; Morris et al. 1994; Zambias et al. 1992). Even a cilofungin derivative with ʟ-threonine at position **6** showed activity. In another series of experiments with simplified synthetic anidulafungin derivatives, i.e., in which the hydroxylated amino acids were largely substituted by their proteinogenic counterparts, these also proved to be bioactive in vitro when l-proline residues with additional amino groups (*cis*-3, *cis*-4, *cis*-4-aminomethyl) or *cis*-4Hyp were introduced in position **6** (Klein et al. 2000). Even smaller substituents could be attached to a *cis*-4 amino group. A synthetic l-proline derivative (positions **3** and **6**) of cilofungin was reported as active against *Candida* sp. (Zambias et al. 1992). In contrast, moderate to severe reduction of fungicidal activity was found for echinocandin derivatives with unsubstituted l-proline in (position **6**) and (**3**,**6**)-di-l-proline derivatives of cilofungin and anidulafungin (Klein et al. 2000; Zambias et al. 1992). On the other hand, synthetic echinocandin derivatives with unsubstituted l-proline only at position **3** showed even better bioactivity than their 4Hyp counterparts. Incorporation of d-proline at position **6** greatly decreased activity, suggesting that the l-configuration is essential. More recently, it has been shown that an *Aspergillus pachychristatus* mutant which cannot hydroxylate proline only produced biologically inactive echinocandin B derivatives (Zhang et al. 2018).

The importance of pneumocandin B_0_ production and the general interest in the complex hydroxyproline system in echinocandin biosynthesis has led to a number of biosynthetic studies, especially with *G. lozoyensis* and *Aspergillus pachychristatus*, the producers of pneumocandins and echinocandin B, respectively. Of all tested pneumocandins, pneumocandin B_0_ had the best bioactivity profile (Schmatz et al. 1992). Unfortunately, the wild-type strain produces this *trans*-3Hyp variant of pneumocandin A_0_ (4Me3Hyp at position **6**) only as a minor fraction (A_0_/B_0_ = 7:1). Through several rounds of classical mutagenesis, *G. lozoyensis* variants with modified product spectrum were generated, of which strain *G. lozoyensis* ATCC 74030 produced pneumocandin B_0_ with a selectivity of A_0_/B_0_ = 1:80 without loss of overall productivity (Masurekar et al. 1992). In parallel, ^13^C-labeling experiments with the wild-type strain revealed that 3Hyp and 4Hyp are biosynthetically derived from l-proline, while 4Me3Hyp originates from l-leucine, which must have been cyclized oxidatively (Adefarati et al. 1991; Adefarati et al. 1992).

About one decade later, Petersen et al. (2003) identified an α-ketoglutarate (αKG)-dependent proline hydroxylase activity in protein crude extracts of *G. lozoyensis* ATCC 74030 that hydroxylated l-proline to 4Hyp and, to a smaller extent, to 3Hyp. The corresponding enzymes have been found later through genomic approaches. Yet, the first enzyme of the hydroxyproline branch of echinocandin biosynthesis described at the molecular level was the expected leucine dioxygenase EcdK (LDO in Fig. 2) from *A. pachycristatus* (Jiang et al. 2013). Although the activity of the heterologously produced αKG-dependent enzyme was very low (5–6 turnovers before inactivation), an iterative hydroxylation and oxidation to the aldehyde followed by spontaneously cyclization to pyrroline carboxylate could be shown. The pyrroline is then reduced to *trans*-4-methyl-l-proline by an enzyme which is not included in the echinocandin biosynthetic gene clusters. Notably, a Δ^1^-pyrroline-5-carboxylate reductase from *G. lozoyensis* has been characterized as early as 1996 (Shyadehi et al. 1996). Deletion of EcdK in *A. pachychristatus* abolished echinocandin production, suggesting that *trans*-4-methylproline or rather 4Me3Hyp are essential for echinocandin B biosynthesis.

The genomes of the pneumocandin A_0_ producer *G. lozoyensis* wild-type (ATCC 20868) and pneumocandin B_0_ production strain ATCC 74030 have been sequenced independently (Chen et al. 2013; Youssar et al. 2012), which allowed the identification of point mutations. In fact, the leucine dioxygenase (GLOXY4 in wild-type ATCC 20868, equivalent to GloC in mutant ATCC 74030) is the only affected enzyme in strain ATCC 74030. Thus, methylproline biosynthesis and not proline hydroxylation caused the changes in the pneumocandin product spectrum (Chen et al. 2015). Through targeted deletion of GLOXY4 in the wild-type *G. lozoyensis*, a mutant was created that was unable to produce pneumocandin A_0_. However, the loss of pneumocandin A_0_ was more than compensated by a 9.5-fold increase of pneumocandin B_0_ production (49 mg L^−1^).

The proline hydroxylase from pneumocandin biosynthesis was originally discovered through screening of the heterologously produced αKG-dependent dioxygenases of the pneumocandin biosynthetic gene cluster in *G. lozoyensis* with putative amino acid substrates (Houwaart et al. 2014). Only one activity was detected: GloF catalyzed the hydroxylation of l-proline to *trans*-4- and *trans*-3-Hyp in an approximate ratio of 8:1, the same ratio as needed by wild-type *G. lozoyensis* for the 7:1 production of pneumocandin A_0_ and B_0_. All these findings suggest that in pneumocandin biosynthesis, Hyp incorporation at position **6** is largely driven by the availability of Hyp substrates rather than a distinct substrate selectivity of the NRPS adenylation domain (A-domain). This is different in echinocandin B biosynthesis by *A. pachychristatus*: here, the proline hydroxylase HtyE produces even more 3Hyp (3Hyp/4Hyp ≈ 1:2.5–3.0; (Mattay et al. 2018; Zhang et al. 2018)). However, 3Hyp is not found in echinocandin products. The idea of a strictly 4Me3Hyp-specific A domain in module **6** is also supported by the fact that the disruption of leucine dioxygenase (EcdK) in *A. pachychristatus* abolished echinocandin production (Jiang et al. 2013), while a knock out in *G. lozoyensis* (GLOXY4) did not impair the overall pneumocandin production. Notably, the differences of the A-domains could also be demonstrated on the structure level: a comparison of homology models of the 6 A-domains showed that the substrate binding pocket is more compact in *A. pachychristatus* and therefore likely more specific (Chen et al. 2015).

Apart from GloF and HtyE, the proline hydroxylase form aculeacin A biosynthesis in *A. aculeatus* NRRL 5994 (Aa-HtyE) has been characterized through heterologous expression in *Escherichia coli* (Zhang et al. 2018). Aculeacin biosynthesis is closely related to echinocandin B biosynthesis; 4Me3Hyp is found exclusively at position **6**. Aa-HtyE produces *trans*-3- and *trans*-4-Hyp in a ratio of 1:7.2. To investigate the structural basis of the enzyme’s selectivity, a meaningful homology model of the active site could be generated, even though the template (anthocyanidin synthase from *Arabidopsis thaliana*; PDB, 1GP6) shared only 22% sequence identity with Aa-HtyE. By superposition of the active sites of the model with a structure of bacterial *cis-*4-prolinehydroxylase (PDB, 4P7X), again a protein unrelated to fungal prolinehydroxylases, substrate, and cosubstrate could be transferred into the model. Based on this, site-directed mutagenesis in proximity to the active site of HtyE showed effects. A double variant was identified in which the *trans*-3-/*trans*-4 selectivity was altered from 1:2.5 to 1:11.0 without loss of activity with native substrates, l-proline and *trans*-4-methyl-l-proline. In contrast, a significant shift towards an increased production of 3Hyp was not found in the screening. In the same study, the disruption of prolinehydroxylase HtyE in *A. pachychristatus* is reported. LC-MS analysis showed that the mutant strain produced products corresponding to echinocandin B and C minus two hydroxyl groups. The (supposed) products are the first biosynthetic echinocandins without 4Hyp at position **3**. They were not bioactive in *Candida* plate assays (Yue et al. 2015), which is in principle consistent with earlier findings discussed above that echinocandins with unsubstituted prolines, especially at position **3**, show poor bioactivity.

The preferred amino acid at position **4** is (3*S*,4*S*)-3,4-dihydroxy-l-homotyrosine. Several variants without side chain hydroxylation at C-3 and particularly at C-4 are known. In addition, the aromatic hydroxyl group may be sulfated or an additional sulfate group is present next to it (Table S3). Structure−activity investigations with partly simplified synthetic echinocandins revealed that, as for other residues, the side chain hydroxylation is not essential for bioactivity (Klein et al. 2000; Zambias et al. 1992). However, the longer side chain of homotyrosine compared to tyrosine is essential. The ethylene unit may be replaced by an ether unit (–CH_2_–O–CH_2_– instead of –CH_2_–CH_2_–) but not a methylene group as in tyrosine. The phenolic hydroxyl group is also important. It can be replaced by amino or phosphate groups in synthesized model compounds without significant loss of bioactivity. For pneumocandin B_0_ phosphate, an in vivo dephosphorylation to the active compound was assumed, so that it has been considered as a water-soluble prodrug (Balkovec et al. 1992). Other substituents at this position, i.e., acylation products, hydrogen, methyl, and hydroxymethyl significantly reduced bioactivity (Balkovec et al. 1992; Klein et al. 2000). A strongly deactivating effect was also found for d-configured homotyrosine. ^13^C-Labeling experiments have shown that homotyrosine is biosynthesized from ʟ-tyrosine and acetate (Adefarati et al. 1991). A more detailed pathway could be derived from the (putative) enzymes encoded in the biosynthetic gene cluster from *A. pachychristatus (*Cacho et al. 2012*)*, which are homologous to those in a fungal pathway for the interconversion of isopropylvalerate (the ketoform of valine) into l-leucine (Fig. 2A) (reviewed in Kohlhaw 2003): First, an isopropylmalate synthase–like enzyme adds acetyl-CoA to *para*-hydroxyphenylpyruvate (the ketoform of tyrosine). The product is isomerized by an aconitase and decarboxylated by a dehydrogenase to the α-ketoacid, which is then transaminated to ʟ-homotyrosine. The subsequent hydroxylations are catalyzed by an αKG-dependent dioxygenase (at C-3) and a CYP-monooxygenase (at C-4): The αKG-dependent enzyme from *A. pachychristatus* (EcdG) was overproduced in *E. coli* and hydroxylated-free homotyrosine at C-3, albeit with very low productivity (3–3.5 turnovers before inactivation). Homotyrosine derivatives of echinocandin B were not converted, so that a hydroxylation before assembly into the echinocandin peptide was assumed. The CYP-monooxygenase (HtyF in *A. pachychristatus*) required for hydroxylation at C-4 has not been investigated in the original report; however, HtyF was the only remaining enzyme from the biosynthetic gene cluster that might catalyze this reaction. In addition, the *Colephorma cylidospora* strains no. 14573 and FERM:BP-5796 do not encode orthologs of HtyF in their clusters and, accordingly, do not produce echinocandins with 4-hydroxyhomotyrosine (cf. Supporting information) (Hino et al. 2001; Kanasaki et al. 2006; Wingfield et al. 2018). Disruption of homotyrosine C-3 and also ornithine hydroxylation in *A. pachychristatus* also reduced homotyrosine C-4 hydroxylation. Hence, a fully hydroxylated substrate is preferred by homotyrosine C-4 hydroxylase. This also implies that C-4-hydroxylation is the last or one of the last steps in echinocandin biosynthesis. In some echinocandins, the phenolic hydroxyl group is sulfated, or a sulfate group has been introduced oxidatively in *ortho* position to the hydroxy group. The genes of the associated enzymes are not included in the biosynthetic gene clusters. As sulfation drastically increases the water solubility of echinocandins, such a group adjacent to the phenol group is very advantageous for the application of an echinocandin, e.g., in micafungin (Fujie 2007).

Position **5** is occupied by (3*S*)-3-hydroxyglutamine in echinocandins from Leotiomycetes (Helotiales) and ʟ-threonine in echinocandins from Eurotiomycetes (Aspergillaceae). An exception is acrophiarin biosynthesis in *P. arenicola*, which possesses a hybrid biosynthetic gene cluster (see above, (Lan et al. 2020)). Other structural exceptions are ʟ-glutamine in cryptocandin produced by *Cryptosporiopsis* cf. *quercina* and ʟ-serine in mulundocandins from *Aspergillus mulundensis* (Table S3). The natural variability of this residue already indicates a relaxed structure−activity relation. Substitutions with ʟ-serine, ʟ-ethyleneglycine, glycine, ʟ-glutamate, ʟ-diaminopropionic acid, and ʟ-arginine in simplified echinocandins retained bioactivity (Klein et al. 2000). With ʟ-tyrosine, the bioactivity was moderately reduced, while with d-threonine and particularly α,α-dimethylglycine, it was strongly impaired. The synthetic reduction of the 3-hydroxy-l-glutamine residue in pneumocandin B_0_ to 3-hydroxy-l-ornithine is advantageous, as it improves both bioactivity and water solubility (Bouffard et al. 1994) and is therefore applied in caspofungin semisynthesis.

The non-proteinogenic 3-hydroxyglutamine is biosynthesized through hydroxylation of ʟ-glutamine (or possibly ʟ-glutamate) by an (putative) αKG-dependent glutamine hydroxylase. A candidate gene is found in all known biosynthetic gene clusters from Leotiomycetes but not those evolved in Eurotiomycetes, and therefore, a function can be clearly inferred. However, gene deletions are not reported, and the corresponding protein GloE (=GLOXY3*)* from *G. lozoyensis* produced in *E. coli* showed no activity with ʟ-glutamine or ʟ-glutamate. Surprisingly, evidence of the function of this enzyme came recently from an evolutionary distant species. *GloE* has been successfully applied to trace the ibotenic acid biosynthetic gene cluster in the genome of the basidiomycete *Amanita muscaria* (fly agaric) (Obermaier and Müller 2020). The homologous protein IboH from *A. muscaria* shared ∼ 42% sequence identity with GloE, and therefore, the identification was comparatively clear. IboH heterologously produced in *E. coli* hydroxylated ʟ-glutamate, but no ʟ-glutamine, to form the (*R*)-3-hydroxylated product.

## Conclusion

Due to their enormous medical importance, the echinocandins are one of today’s best-studied non-ribosomal peptide natural product families. Initially by purely industrial research, it was not only possible to optimize production and metabolite spectrum, but also to elucidate the biosynthesis. In the age of genomics, the biomolecular basics could finally be explored. Nevertheless, research on echinocandins faces further challenges. Only some of the enzymes and proteins are sufficiently characterized to describe the biosynthetic process in detail. Synthetic biology approaches will be required for the economic production of tailor-made echinocandin antifungals. First advances, mostly based on targeted gene deletion, have already been made (Chen et al. 2016; Chen et al. 2015; Li et al. 2015). However, the key enzyme for the assembly of echinocandins is the huge NRPS, whose A domains, which are responsible for substrate specificity, are of particular interest. The ten currently known orthologous NRPS provide a good basis for comparative investigations in this complex field. In addition, it would be very helpful for the development of echinocandins, to reveal the structure of the target of echinocandins, the 1,3-β-glucan synthase.

In parallel to the development of antimycotics, echinocandin biosynthesis also opens doors for the biosynthesis of new synthetic building blocks, including a branched fatty acid (dimethyl myristate) and two amino acids with an unusual carbon skeleton (homotyrosine and *trans*-4-methyl proline). These and other amino acids can be modified with the in total six hydroxylases. However, not only the biotechnological aspect is of importance; with its well-defined biosynthetic clusters, echinocandin biosynthesis is an excellent model for the study of gene clusters and their evolution in fungi (Yue et al. 2015). Finally, the knowledge gained from echinocandin biosynthesis can even lead to unexpected findings, such as the discovery of ibotenic acid biosynthesis in the fly agaric (*A. muscaria)* (Obermaier and Müller 2020)

## Supplementary information

ESM 1 (PDF 412 kb)
