# Supplementary material for: Echinocandins: structural diversity, biosynthesis, and development of antimycotics
Source: Appl Microbiol Biotechnol. 2020 Dec 3;105(1):55–66. doi: 10.1007/s00253-020-11022-y (PMC7778625; doi:10.1007/s00253-020-11022-y)
Supplement: Supplementary file 1 — (PDF 412 kb) [file 253_2020_11022_MOESM1_ESM.pdf]

Supplementary Materials

Applied Microbiology and Biotechnology

Echinocandins: Structural diversity, Biosynthesis and Development of Antimycotics

Wolfgang Hüttel

Institute of Pharmaceutical Sciences

University of Freiburg

Albertstr. 25

79106 Freiburg, Germany

E-mail: wolfgang.huettel@pharmazie.uni-freiburg.de

Tel.: (+49) 0761/203-67398

Fax: (+49) 0761/203-6351

A) Biosynthetic gene clusters

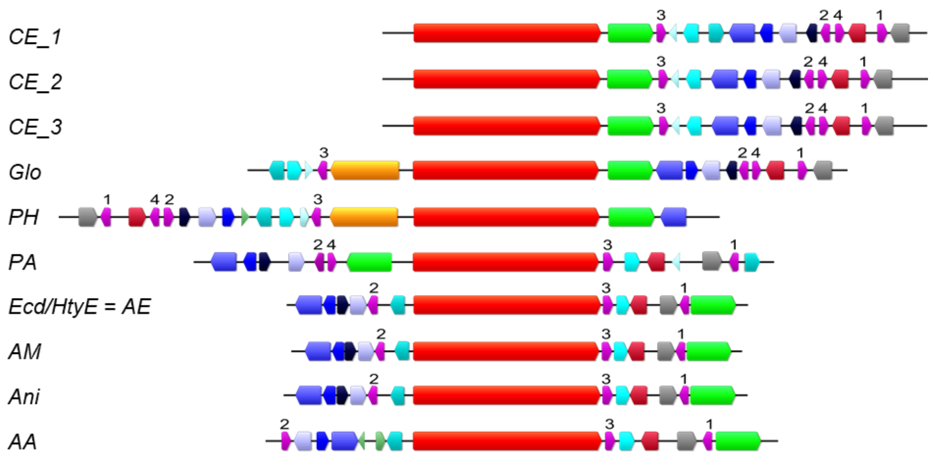

Figure S1: Maps of echinocandin biosynthetic gene clusters. The organisms are listed in Tables S1, and the color code of genes is explained in Table S2.

Table S1. Echinocandin biosynthetic gene clusters.

Cluster	Organism	NCBI-ID
CE_1	*Coleophoma cylindospora* F-11899, FERM:BP-2635 (previously *C. empetri)*	AB723722
CE_2	*Coleophoma cylindospora* No. 14573, FERM:BP-6252 (previously *C. empetri)*	AB720725
CE_3^{CC}	*Coleophoma cylindospora* FERM:BP-5796 (previously *C. crateriformis)*	AB720076
GL/Glo	GL: *Glarea lozoyensis* wild-type strain ATCC 20868<br>Glo: *Glarea lozoyensis* mutant strain ATCC 74030	NW007360987<br>AGUE01000179
PH	*Phialophora* cf. *hyalina* No. 16616 (previously *Tolypocladium parasiticum*)	AB720726
PA	*Penicillium arenicola* NRRL 8095 (suggested genus: *Phialomyces*; previously *Acrophialophora limonispora* nov. spec.)	MN518690
Ecd/Hty<br>=<br>*AE*	*Ecd/Hty*: *Aspergillus pachycristatus* NRRL 11440 (previously *Emericella rugulosa*)<br>[virtually identical with the *AE* cluster, originally deposited as a sequence from *Aspergillus spinulosporus* NRRL 3860, (previously *Aspergillus delacroxii* and *Emericella nidulans* var. *echinulatus*)](Hüttel et al. 2016)	alignment of *Ecd*: JX421684 and *Hty*: JX421685<br>*AE*: AB720074
AM	*Aspergillus mulundensis* DSMZ 5745	KP742486
Ani	*Aspergillus nidulans* NRRL 8112	KT806042
AA	*Aspergillus aculeatus* ATCC 16872	GOLD Project ID: Gp0010055

Table S2: Proteins encoded in echinocandin biosynthetic gene clusters.

**Color**	**Name**	**Protein function**	**Remarks**
	NRPS	nonribosomal peptide synthetase	
	MDT	multidrug transporter	
	FAC	long-chain fatty acyl-CoA ligase	
	IPMS	isopropylmalate synthase	homotyrosine biosynthesis
	ACN	aconitase	homotyrosine biosynthesis
	IPMDH	isopropylmalate dehydrogenase	homotyrosine biosynthesis
	TA	transaminase	homotyrosine biosynthesis, not in cluster *AA*
	P1	putative protein, unknown function	
**1**	LDO	αKG-dependent L-leucine dioxygenase	very low in vitro activity
**2**	PH	αKG-dependent L-proline hydroxylase	in vitro activity
**3**	hT3H	αKG-dependent L-homotyrosine 3-hydroxylase	very low in vitro activity
**4**	GH	αKG-dependent putative L-glutamine hydroxylase	*Leotiomycetes* clusters and cluster *PA*
	OrnH	CYP, L-ornithine 4,5-hydroxylase	
	hT4H	CYP, putative L-homotyrosine 4-hydroxylase	absent in cluster *CE_2* and *CE_3*
	ABH	putative thioesterase (α/β-hydrolase)	*Leotiomycetes* clusters and cluster *PA*
	PKS	polyketide synthase	*Leotiomycetes* clusters

B) Compounds and producer strains

Table S3: Echinocandins isolated from wildtype and mutant strains.

[a] Main products of wildtype strains are shown in bold. [*] Only known from mutant strain. [b] Differences from the archetype pneumocandin A_0 are indicated in bold. [c] Characterized only by LC-MS techniques. [d] proposed name. For an overview on “FR” compounds, see Hashimoto (2009); for “WF” classification see Hino et al. (2001). Revised and updated from (Hüttel 2017).

Amino acid abbreviations:

<u>Position 1</u>: *Orn*: L-ornithine; dh*Orn*: (4*R*,5*R*)-4,5-dihydroxy-L-ornithine; 4h*Orn*: (*R*)-4-hydroxy-L-ornithine; 5h*Orn*: (*R*)-5-hydroxy-L-ornithine.

<u>Positions 3 and 6:</u> 4-*Hyp*: *trans*-4-hydroxy-L-proline, 3-*Hyp*: *trans*-3-hydroxy-L-proline; 4Me3*Hyp*: (3*S*,4*S*)-3-hydroxy-4-methylproline; 4hMe3*Hyp*: (3*S*,4*R*)-3-hydroxy-4-hydroxymethyl-L-proline; 3,4d*Hyp*: (3*R*,4*S*)-3,4-dihydroxy-L-proline.

<u>Position 4:</u> *hTyr*: L-homotyrosine; 3,4dh*hTyr*: (3*S*,4*S*)-3,4-dihydroxyhomotyrosine; 3h*hTyr*: (*R*)-3-hydroxy-L-homotyrosine; 4h*hTyr*: (*R*)-4-hydroxy-L-homotyrosine; -3'SO_4: sulfate group at aromatic position 3' of the specified homotyrosine; -4-OH: the phenol group at 4' of the specified homotyrosine is sulfated; -3'OH,4'SO_4: the phenol is sulfated at 4' and hydroxylated at 3'.

<u>Position 5:</u> h*Gln*: (*R*)-3-hydroxy-L-glutamine.

**Abbr.**	**Compound**[a] (Synonyms)	**Fatty acid chain**	**Amino acid:**[b] **1**[	**2**	**3**	**4**]	**5**]	**6**[
**P_{A_0}**	**Pneumocandin A_0** (L-671,329)	dimethylmyristoyl	dh*Orn*	*Thr*	*4Hyp*	dh*hTyr*	h*Gln*	4Me3*Hyp*
**P_{A_1}**	Pneumocandin A_1	dimethylmyristoyl	dh*Orn*	*Thr*	*4Hyp*	**4h*hTyr***	h*Gln*	4Me3*Hyp*
**P_{A_2}**	Pneumocandin A_2	dimethylmyristoyl	***Orn***	*Thr*	*4Hyp*	dh*hTyr*	h*Gln*	4Me3*Hyp*
**P_{A_3}**	Pneumocandin A_3	dimethylmyristoyl	**5h*Orn***	*Thr*	*4Hyp*	***hTyr***	h*Gln*	4Me3*Hyp*
**P_{A_4}**	Pneumocandin A_4	dimethylmyristoyl	***Orn***	*Thr*	*4Hyp*	***hTyr***	h*Gln*	4Me3*Hyp*
**P_{A_G}**	Pneumocandin G* (= Pneumocandin A_G[d])	dimethylmyristoyl	dh*Orn*	*Thr*	*4Hyp*	**3h*hTyr***	h*Gln*	4Me3*Hyp*
**P_{A_9}**	Compound 9* (= Pneumocandin A_9[d])	dimethylmyristoyl	**5h*Orn***	*Thr*	*4Hyp*	***hTyr***	h*Gln*	4Me3*Hyp*
**$P_{A_{13}}$**	Compound 13* (= Pneumocandin A_{13}[d])	dimethylmyristoyl	4h*Orn*	*Thr*	*4Hyp*	***hTyr***	h*Gln*	4Me3*Hyp*
**P_{B_0}**	Pneumocandin B_0	dimethylmyristoyl	dh*Orn*	*Thr*	*4Hyp*	dh*hTyr*	h*Gln*	**3*Hyp***
**P_{B_1}**	Pneumocandin B_1	dimethylmyristoyl	dh*Orn*	*Thr*	*4Hyp*	**4h*hTyr***	h*Gln*	**3*Hyp***
**P_{B_2}**	Pneumocandin B_2	dimethylmyristoyl	***Orn***	*Thr*	*4Hyp*	dh*hTyr*	h*Gln*	**3*Hyp***
**P_{B_5}**	Pneumocandin B_5	dimethylmyristoyl	**5h*Orn***	*Thr*	*4Hyp*	dh*hTyr*	h*Gln*	**3*Hyp***
**P_{B_6}**	Pneumocandin B_6	dimethylmyristoyl	4h*Orn*	*Thr*	*4Hyp*	dh*hTyr*	h*Gln*	**3*Hyp***
**P_{B_F}**	Pneumocandin F* (Pneumocandin B_F)	dimethylmyristoyl	dh*Orn*	*Thr*	*4Hyp*	**3h*hTyr***	h*Gln*	**3*Hyp***
**$P_{B_{10}}$**	Compound 10* (Pneumocandin B_{10}[d])	dimethylmyristoyl	**5h*Orn***	*Thr*	*4Hyp*	***hTyr***	h*Gln*	**3*Hyp***
**$P_{B_{11}}$**	Compound 11* (Pneumocandin B_{11}[d])	dimethylmyristoyl	4h*Orn*	*Thr*	*4Hyp*	***hTyr***	h*Gln*	**3*Hyp***
**$P_{B_{14}}$**	Compound 14* (Pneumocandin B_{14}[d])	dimethylmyristoyl	***Orn***	*Thr*	*4Hyp*	***hTyr***	h*Gln*	**3*Hyp***
**$P_{B_{0\text{-}Ser}}$**	Pneumocandin $B_{0\text{-}Ser}$	dimethylmyristoyl	dh*Orn*	***Ser***	*4Hyp*	dh*hTyr*	h*Gln*	**3*Hyp***
**$P_{B_{5\text{-}Ser}}$**	Pneumocandin $B_{5\text{-}Ser}$	dimethylmyristoyl	**5h*Orn***	***Ser***	*4Hyp*	dh*hTyr*	h*Gln*	**3*Hyp***

**P_{C_0}**	Pneumocandin C_0	dimethylmyristoyl	dh*Orn*	*Thr*	*4Hyp*	dh*hTyr*	h*Gln*	**4*Hyp***
**P_{D_0}**	Pneumocandin D_0	dimethylmyristoyl	dh*Orn*	*Thr*	*4Hyp*	dh*hTyr*	h*Gln*	**3,4d*Hyp***
**P_{D_2}**	Pneumocandin D_2	dimethylmyristoyl	***Orn***	*Thr*	*4Hyp*	dh*hTyr*	h*Gln*	**3,4d*Hyp***
**P_{E_0}**	Pneumocandin E_0	dimethylmyristoyl	dh*Orn*	*Thr*	*4Hyp*	dh*hTyr*	h*Gln*	***Pro***
**P_H**	Pneumocandin H*	myristoyl	dh*Orn*	*Thr*	*4Hyp*	dh*hTyr*	h*Gln*	**3*Hyp***
**P_I**	Pneumocandin I*	pentadecanoyl	dh*Orn*	*Thr*	*4Hyp*	dh*hTyr*	h*Gln*	4Me3*Hyp*
**P_J**	Pneumocandin J*	palmitoyl	dh*Orn*	*Thr*	*4Hyp*	dh*hTyr*	h*Gln*	**3*Hyp***
**P_K**	Pneumocandin K*	palmitoyl	dh*Orn*	*Thr*	*4Hyp*	dh*hTyr*	h*Gln*	4Me3*Hyp*
**A**	**Acrophiarin** (Antibiotic 31794/F-1)	**myristoyl**	dh*Orn*	*Thr*	*4Hyp*	dh*hTyr*	h*Gln*	4Me3*Hyp*
**S_A**	**Sporiofungin A** (S 41062/F-1)	dimethylmyristoyl	dh*Orn*	***Ser***	*4Hyp*	**3h*hTyr***	h*Gln*	4Me3*Hyp*
**S_B**	Sporiofungin B (S 41062/F-6)	dimethylmyristoyl	dh*Orn*	*Thr*	*4Hyp*	**3h*hTyr***	h*Gln*	**3*Hyp***
**S_C**	Sporiofungin C (S 41062/F-7)	dimethylmyristoyl	**Medh*Orn***	***Ser***	*4Hyp*	**3h*hTyr***	h*Gln*	4Me3*Hyp*
**C**	**Cryptocandin**	**palmitoyl**	dh*Orn*	*Thr*	*4Hyp*	dh*hTyr*	***Gln***	**4hMe3*Hyp***
**FR_{901379}**	**FR901379** (WF11899A)	**palmitoyl**	dh*Orn*	*Thr*	*4Hyp*	dh*hTyr*-**3'SO_4**	h*Gln*	4Me3*Hyp*
**FR_{901381}**	FR901381 (WF11899B)	**palmitoyl**	dh*Orn*	*Thr*	*4Hyp*	**3h*hTyr*-3'SO_4**	h*Gln*	4Me3*Hyp*
**FR_{901382}**	FR901382 (WF11899C)	**palmitoyl**	**5h*Orn***	*Thr*	*4Hyp*	**3h*hTyr*-3'SO_4**	h*Gln*	4Me3*Hyp*
**FR_{220897}**	**FR220897** (WF14573B)	**palmitoyl**	dh*Orn*	*Thr*	*4Hyp*	**3h*hTyr*-3'OH,4'SO_4**	h*Gln*	4Me3*Hyp*
**FR_{220897}**	**FR220899** (WF14573A)	**palmitoyl**	dh*Orn*	*Thr*	*4Hyp*	**3h*hTyr*-3'OH,4'SO_4**	h*Gln*	**3*Hyp***
**FR_{190293}**	**FR190293** (WF16616)	dimethylmyristoyl	dh*Orn*	*Thr*	*4Hyp*	dh*hTyr*-**3'SO_4**	h*Gln*	4Me3*Hyp*
**Fr_{227673}**	FR227673 (WF22210)	**dimethylpalmitoyl**	dh*Orn*	*Thr*	*4Hyp*	dh*hTyr*-**3'SO_4**	h*Gln*	4Me3*Hyp*
**FR_{209602}**	**FR209602** (WF738A)	**palmitoyl**	dh*Orn*	***Ser***	*4Hyp*	**3h*hTyr*-3'SO_4**	h*Gln*	4Me3*Hyp*
**FR_{209603}**	FR209603 (WF738B)	**palmitoyl**	dh*Orn*	***Ser***	*4Hyp*	**3h*hTyr*-3'SO_4**	h*Gln*	**3*Hyp***
**FR_{209604}**	FR209604 (WF738C)	**palmitoyl**	**5h*Orn***	***Ser***	*4Hyp*	**3h*hTyr*-3'SO_4**	h*Gln*	4Me3*Hyp*
**E_B**	**Echinocandin B** (A-30912 Factor A, Antibiotic A-22082, SL7810/F)	**linoleoyl**	dh*Orn*	*Thr*	*4Hyp*	dh*hTyr*	***Thr***	4Me3*Hyp*
**E_C**	Echinocandin C (A-30912 Factor B, SL 7810/F-II)	**linoleoyl**	dh*Orn*	*Thr*	*4Hyp*	**3h*hTyr***	***Thr***	4Me3*Hyp*
**E_D**	Echinocandin D (A-30912 Factor D, SL 7810/F-III)	**linoleoyl**	***Orn***	*Thr*	*4Hyp*	**3h*hTyr***	***Thr***	4Me3*Hyp*
**$E_{B_{Pro}}$**	Echinocandin B_{Pro}*[c]	**linoleoyl**	dh*Orn*	*Thr*	***Pro***	dh*hTyr*	***Thr***	**4MePro**
**$E_{C_{Pro}}$**	Echinocandin C_{Pro}*[c]	**linoleoyl**	dh*Orn*	*Thr*	***Pro***	**3h*hTyr***	***Thr***	**4MePro**
**E_{De1}**	Echinocandin B derivative 1*[d]	**linoleoyl**	***Orn***	*Thr*	*4Hyp*	dh*hTyr*	***Thr***	4Me3*Hyp*
**E_{De2}**	Echinocandin B derivative 2*[d]	**linoleoyl**	dh*Orn*	*Thr*	*4Hyp*	**4h*hTyr***	***Thr***	4Me3*Hyp*
**E_{De3}**	Echinocandin B derivative 3*[d]	**linoleoyl**	dh*Orn*	*Thr*	*4Hyp*	***hTyr***	***Thr***	4Me3*Hyp*
**E_{De4}**	Echinocandin B derivative 4*[d]	**linoleoyl**	**5h*Orn***	*Thr*	*4Hyp*	***hTyr***	***Thr***	4Me3*Hyp*
**E_{De5}**	Echinocandin B derivative 5*[d]	**linoleoyl**	***Orn***	*Thr*	*4Hyp*	***hTyr***	***Thr***	4Me3*Hyp*
**A_{30912H}**	A-30912 Factor H	**linoleoyl**	**Medh*Orn***	*Thr*	*4Hyp*	dh*hTyr*	***Thr***	4Me3*Hyp*
**A_{A_y}**	**Aculeacin A or A_y**	**palmitoyl**	dh*Orn*	*Thr*	*4Hyp*	dh*hTyr*	***Thr***	4Me3*Hyp*
**A_{A_α}**	Aculeacin A_α	**myristoyl**	dh*Orn*	*Thr*	*4Hyp*	dh*hTyr*	***Thr***	4Me3*Hyp*

**A_{D_γ}**	Aculeacin D_γ	**palmitoyl**	dh*Orn*	*Thr*	*4Hyp*	**3h*hTyr***	***Thr***	4Me3*Hyp*
**A_{D_α}**	Aculeacin D_α	**myristoyl**	dh*Orn*	*Thr*	*4Hyp*	**3h*hTyr***	***Thr***	4Me3*Hyp*
**M**	**Mulundocandin**	**12-methylmyristoyl**	dh*Orn*	*Thr*	*4Hyp*	dh*hTyr*	***Ser***	4Me3*Hyp*
**dM**	Deoxymulundocandin	**12-methylmyristoyl**	dh*Orn*	*Thr*	*4Hyp*	**3h*hTyr***	***Ser***	4Me3*Hyp*

Table S4: Fungal strains producing echinocandins. Revised and updated from Hüttel (2017).

Species	Metabolites	Strain (Comment)
**a) Wild-type strains**		
Penicillium arenicola (=Acrophialophora Iimonispora nov. spec. 'Dreyfuss + Muller'; suggested genus: *Phialomyces*)	A	NRRL 8095 (cluster Pa)(Dreyfuss 1986; Dreyfuss and Tscherter 1979; Lan et al. 2020), additional producer strains: NRRL 3392, NRRL 31507, NRRL 31509 (Lan et al. 2020)
Aspergillus aculeatus	$\mathbf{A_{Ay}}$ $A_{A\alpha}$,, $A_{D\gamma}$, $A_{D\alpha}$	M-4845, NRRL 11270 (Otani et al. 1978)
	A_{Ay} and others	M-4214, NRRL 8075 (Mizuno et al. 1977; Satoi et al. 1977)
Aspergillus aculeatus (= *A. japonicus* var. *aculeatus*)	A_{Ay}	ATCC 16872, NRRL 5094 (cluster: *AA* (lacks aminotransferase AT) genome available under GOLD seq. ID: Gp0010055) (Nordberg et al. 2014)
Aspergillus mulundensis (= *A. sydowii* Thorn and Church var. nov. *mulundensis* Roy)	**M,** dM	Cult. No. Y-30462, DSMZ 5745 (cluster: *AM)* (Bills et al. 2016; Mukhopadhyay et al. 1987; Roy et al. 1987; Wingfield et al. 2018; Yue et al. 2015)
Aspergillus nidulans	E_B	NRRL 8112, ATCC 58396 (cluster: *Ani*) (Higgens and Michel 1977)
	E_B	ZBJ-0817, CCTCC M 2010275 (Zou et al. 2015)
	E_B	F-109,962 (de la Cruz et al. 2012)
	E_B	F-167,724 (de la Cruz et al. 2012)
	E_B	CBS 240.9 (de la Cruz et al. 2012)
	E_B	CBS 542.83 (de la Cruz et al. 2012)
Aspergillus pachycristatus (= *Emericella rugulosa*, *A. nidulans* var. *roseus*)	$\mathbf{E_B}$, $E_{C,}$ $E_{D,}$ A_{30912^H}	NRRL 11440, ATCC 58397 (products: "*A-42355 antibiotic mixture*"; cluster: *Ecd*/*Hty* (Boeck and Kastner 1981; Lan et al. 2019; Yue et al. 2015)
Aspergillus rugulosus	$\mathbf{E_B}$, $E_{C,}$ $E_{D,}$ an others	NRRL 8113 (Factors A–G) (Hoehn and Michel 1977)
	$\mathbf{E_B}$, $E_{C,}$ $E_{D,}$	NRRL 8039 (Keller-Juslén et al. 1976; Traber et al. 1979), further strains are listed in Dreyfuss (1986)
Aspergillus spinulosporus NRRL 3860 (=A. delacroxii, Emericella nidulans var. *echinulata)*	E_B	A 32204, NRRL 3860 (stated cluster: *AE is identical with Ecd*/*Hty* (Benz et al. 1974)
Chalara sp. (= *Pochonia parasitica*, *Tolypocladium parasiticum*)	Fr_{227673}	FERM:BP-5553, No. 22210 (Hino et al. 2001; Kanasaki et al. 2006b)
Coleophoma cylindospora (= *C. crateriformis*)	$\mathbf{FR_{209602}}$, FR_{209603}, FR_{209604}	FERM:BP-5796, No. 738, cluster: CC*_1*, *prfks1a* gene (Kanasaki et al. 2006c; Wingfield et al. 2018)
(= *C. empetri*)	$\mathbf{FR_{901379}}$, FR_{901381}, FR_{901382}	FERM:BP-2635, F-11899 (cluster: CC_2*CE–1*) (Iwamoto et al. 1994)
(= *C. empetri*)	$\mathbf{FR_{220897}}$, $\mathbf{FR_{220899}}$	FERM:BP-6252, No. 14573, cluster: CC_3*CE–2*, *prfks1a* gene (Hino et al. 2001; Kanasaki et al. 2006a; Wingfield et al. 2018)
Cryptosporiopsis cf. *quercina*	C	(Strobel et al. 1999)
Cryptosporiopsis sp.	P_{A0}	"isolate P30A" (Noble et al. 1991)
Emericella cleistominuta	E_B	CBS 200.75 (de la Cruz et al. 2012)
Emericella navahoensis	E_B	CBS 351.81 (de la Cruz et al. 2012)
Emericella parvathecia	E_B	CBS 493.65 (de la Cruz et al. 2012)
Emericella quadrilineata	E_B	J. Guarro WD-007 (de la Cruz et al. 2012)
	E_B	CBS 114511 (de la Cruz et al. 2012)
	E_B	J. Guarro WH-042 (de la Cruz et al. 2012)
Emericella rugulosa	E_B	J. Guarro WO-021 (de la Cruz et al. 2012)
	E_B	J. Guarro WO-148 (de la Cruz et al. 2012)
	E_B	CBS 171.71 (de la Cruz et al. 2012)
	E_B	CBS 852.96 (de la Cruz et al. 2012)

	E_B	J. Guarro WP-050 (de la Cruz et al. 2012)
Emericella sp.	E_B	F-166,688 (de la Cruz et al. 2012)
Glarea lozoyensis (= *Zalerion arboricola*)	**P_{A_0}**, P_{A_1}, P_{A_3}, P_{A_4}, **P_{B_0}**, P_{B_1}, P_{B_2}, P_{B_5}, P_{B_6}, $P_{B_0\text{-Ser}}$, $P_{B_5\text{-Ser}}$, P_{C_0}, P_{D_2} (wild-type), P_{E_0}	ATCC 20868, F-160870 (pneumocandin $A_0/B_0 \approx 7:1$; cluster: *GL*) (Bills et al. 1999; Li et al. 2015; Masurekar et al. 1992; Morris et al. 1994a; Peláez et al. 2011; Schwartz et al. 1989; Schwartz et al. 1992)
	**P_{A_0}**, P_{A_1}, P_{B_0}	F-226836 (Peláez et al. 2011)
	**P_{A_0}**, P_{A_1}, P_{B_0}	F- 226838 (Peláez et al. 2011)
	**P_{A_0}**, P_{A_1}, P_{B_0}	F- 239379 (Peláez et al. 2011)
Pezicula sp.	P_{A_0}	(Noble et al. 1991)
Pezicula radicicola (= *Cryptosporiopsis* sp.)	**S_A**, S_B, S_C	NRRL 12192, ATCC 20594, S 41 062/F cluster: *Pr*, *prfks1a* gene (Balkovec 1994; Dreyfuss 1986; Dreyfuss and Tscherter 1982; Yue et al. 2018)
Venustampulla echinocandica (= *Phialophora* cf. *hyaline, Tolypocladium parasiticum*)	Fr_{190293}	No. 16616, FERM:BP-5553, cluster: *Tpa* (Bills et al. 2014; Hino et al. 2001; Kanasaki et al. 2006b; Untereiner et al. 2019; Wingfield et al. 2018)
**b) Mutant strains**		
Glarea lozoyensis	P_{A_0}, **P_{B_0}**, P_{D_0}	ATCC 20957 (pneumocandin $A_0/B_0 \approx 1{:}10$) (Masurekar et al. 1992), (Morris et al. 1994b)
	P_{A_2}	ATCC 20958 (main product: pneumocandin A_2) (Masurekar et al. 1992)
	P_{A_2}, P_{A_4}	ATCC 20988 (main products: pneumocandin A_2 and A_4) (Masurekar et al. 1992)
	P_{A_0}, **P_{B_0}**	ATCC 74030 (pneumocandin $A_0/B_0 \approx 1{:}80$, cluster: *Glo*) (Masurekar et al. 1992)
	P_{B_0}	CGMCC 2933 (Developed from strain ATTC 20957 increased pneumocandin B_0 production, cluster: *Glo*) (Xu et al. 2012)
	P_{A_1}, P_{A_3}, P_{A_4}, P_{A_9}, $P_{A_{13}}$, P_{B_1}, $P_{B_{10}}$, $P_{B_{11}}$, $P_{B_{14}}$	ATCC 20868 ΔGLOXY1 (no hydroxylation at *hTyr* C-3) (Li et al. 2015)
	**P_{B_0}**	ATCC 20868 ΔGLOXY4 (10GL0% B_0) (Chen et al. 2015)
	P_F, P_G	ATCC 20868 ΔGLP450-1 (Li et al. 2015)
	P_{A_2}, P_{B_2},	ATCC 20868 ΔGLP450-2 (Li et al. 2015)
	A, $P_{I\text{–}K}$	ATCC 20868 ΔGLPKS4 (Chen et al. 2016)
Aspergillus nidulans	E_B	ULN-59 (Hu et al. 2016; Zou et al. 2015)
	E_B	ZBJ12037, CCTCC M 2015677 (Hu et al. 2016)
Phialophora cf. *hyalina* (= *Tolypocladium parasiticum*)	FR_{190293}	No. 16616 (increased production of WF16616) (Haifeng et al. 2013)
Aspergillus pachycristatus	E_D, E_{De1}	ATCC 58397 ΔecdH (ornithine hydroxylase disruption)
	E_{De2}, E_{De3}, E_{De4}, E_{De5},	ATCC 58397 ΔecdG (homotyrosine hydroxylase disruption)
	E_{BPro}, E_{CPro}	ATCC 58397 ΔhtyE (proline hydroxylation disruption)

References:

Balkovec JM (1994) Section Review: Anti-infectives: Lipopeptide antifungal agents. Expert Opin Invest Drugs 3(2):65–82 doi:doi:10.1517/13543784.3.2.65.

Benz F, Knüsel F, Nüesch J, Treichler H, Voser W, Nyfeler R, Keller-Schierlein W (1974) Stoffwechselprodukte von Mikroorganismen 143. Mitteilung. Echinocandin B, ein neuartiges Polypeptid-Antibioticum aus *Aspergillus nidulans* var. *echinulatus*: Isolierung und Bausteine. Helv Chim Acta 57(8):2459–2477 doi:10.1002/hlca.19740570818.

Bills G, Li Y, Chen L, Yue Q, Niu X-M, An Z (2014) New Insights into the Echinocandins and Other Fungal Non-Ribosomal Peptides and Peptaibiotics. Nat Prod Rep 31(10):1348–1375 doi:10.1039/C4NP00046C.

Bills GF, Platas G, Peláez F, Masurekar P (1999) Reclassification of a pneumocandin-producing anamorph, *Glarea lozoyensis* gen. et sp. nov., previously identified as *Zalerion arboricola*. Mycol Res 103(2):179–192 doi:http://dx.doi.org/10.1017/S095375629800687X.

Bills GF, Yue Q, Chen L, Li Y, An Z, Frisvad JC (2016) *Aspergillus mulundensis* sp. nov., a new species for the fungus producing the antifungal echinocandin lipopeptides, mulundocandins. J Antibiot 69(3):141–148 doi:10.1038/ja.2015.105.

Boeck LVD, Kastner RE (1981) Method of producing the A-30912 antibiotics. United States Patent US 4288549,

Chen L, Li Y, Yue Q, Loksztejn A, Yokoyama K, Felix EA, Liu X, Zhang N, An Z, Bills GF (2016) Engineering of New Pneumocandin Side-Chain Analogues from Glarea lozoyensis by Mutasynthesis and Evaluation of Their Antifungal Activity. ACS Chem Biol doi:10.1021/acschembio.6b00604.

Chen L, Yue Q, Li Y, Niu X, Xiang M, Wang W, Bills GF, Liu X, An Z (2015) Engineering of *Glarea lozoyensis* for Exclusive Production of the Pneumocandin B_0 Precursor of the Antifungal Drug Caspofungin Acetate. Appl Environ Microbiol 81(5):1550–8 doi:10.1128/AEM.03256-14.

de la Cruz M, Martín J, González-Menéndez V, Pérez-Victoria I, Moreno C, Tormo JR, El Aouad N, Guarro J, Vicente F, Reyes F, Bills GF (2012) Chemical and Physical Modulation of Antibiotic Activity in *Emericella* Species. Chem Biodivers 9(6):1095–1113 doi:10.1002/cbdv.201100362.

Dreyfuss M (1986) Neue Erkenntnisse aus einem pharmakologischen Pilz-Screening. Sydowia 39:22–36

Dreyfuss MM, Tscherter H (1979) Antibiotic S 31794/F-1. United States Patent US 4173629,

Dreyfuss MM, Tscherter H (1982) New metabolites, processes for their production and their use. WIPO Patent WO 1982000587,

Haifeng H, Lijun W, Taoling Y, Baoquan Z, Haifeng H, Lijun W, Taoling Y, Baoquan Z (2013) Method for breeding high-WF16616-yield *Tolypocladium parasiticum* mutant strain. China Patent CN 103087937,

Hashimoto S (2009) Micafungin: a sulfated echinocandin. J Antibiot 62(1):27–35

Higgens CE, Michel KH (1977) Antibiotic A-22082 and Process for Production Thereof. United States Patent US 4024246,

Hino M, Fujie A, Iwamoto T, Hori Y, Hashimoto M, Tsurumi Y, Sakamoto K, Takase S, Hashimoto S (2001) Chemical Diversity in Lipopeptide Antifungal Antibiotics. J Ind Microbiol Biotechnol 27(3):157–162 doi:10.1038/sj.jim.7000091.

Hoehn MM, Michel KH (1977) Antibiotic A-30912. United States Patent US 4024245,

Hu Z-C, Peng L-Y, Zheng Y-G (2016) Enhancement of Echinocandin B Production by a UV- and Microwave-Induced Mutant of *Aspergillus nidulans* with Precursor- and Biotin-Supplying Strategy. Appl Biochem Biotechnol 179(7):1–14 doi:10.1007/s12010-016-2060-5.

Hüttel W (2017) Structural diversity in echinocandin biosynthesis: the impact of oxidation steps and approaches toward an evolutionary explanation. Zeitschrift für Naturforschung C 72(1-2):1 doi:10.1515/znc-2016-0156.

Hüttel W, Youssar L, Grüning BA, Günther S, Hugentobler KG (2016) Echinocandin B biosynthesis: a biosynthetic cluster from *Aspergillus nidulans* NRRL 8112 and reassembly of the subclusters *Ecd* and *Hty* from *Aspergillus pachycristatus* NRRL 11440 reveals a single coherent gene cluster. BMC Genomics 17:570 doi:10.1186/s12864-016-2885-x.

Iwamoto T, Fujie A, Sakamoto K, Tsurumi Y, Shigematsu N, Yamashita M, Hashimoto S, Okuhara M, Kohsaka M (1994) WF11899A, WF11899B and WF11899C, Novel Antifungal Lipopeptides 1. Taxonomy, Fermentation, Isolation and Physicochemical Properties. J Antibiot 47(10):1084–1091

Kanasaki R, Abe F, Kobayashi M, Katsuoka M, Hashimoto M, Takase S, Tsurumi Y, Fujie A, Hino M, Hashimoto S, Hori Y (2006a) FR220897 and FR220899, Novel Antifungal Lipopeptides from *Coleophoma empetri* No. 14573. J Antibiot 59(3):149–157

Kanasaki R, Kobayashi M, Fujine K, Sato I, Hashimoto M, Takase S, Tsurumi Y, Fujie A, Hino M, Hashimoto S, Hori Y (2006b) FR227673 and FR190293, Novel Antifungal Lipopeptides from *Chalara* sp. No. 22210 and *Tolypocladium parasiticum* No. 16616. J Antibiot 59(3):158–167

Kanasaki R, Sakamoto K, Hashimoto M, Takase S, Tsurumi Y, Fujie A, Hino M, Hashimoto S, Hori Y (2006c) FR209602 and Related Compounds, Novel Antifungal Lipopeptides from *Coleophoma crateriformis* No. 738. J Antibiot 59(3):137–144

Keller-Juslén C, Kuhn M, Loosli HR, Petcher TJ, Weber HP, von Wartburg A (1976) Struktur des Cyclopeptid-Antibiotikums sl 7810 (= echinocandin B). Tetrahedron Lett 17(46):4147–4150 doi:http://dx.doi.org/10.1016/S0040-4039(00)74618-1.

Lan N, Perlatti B, Kvitek DJ, Wiemann P, Harvey CJB, Frisvad J, An Z, Bills GF (2020) Acrophiarin (Antibiotic S31794/F-1) from *Penicillium arenicola* Shares Biosynthetic Features with Both *Aspergillus*- and Leotiomycete-Type Echinocandins. Environ Microbiol n/a(n/a) doi:10.1111/1462-2920.15004.

Lan N, Yue Q, An Z, Bills GF (2019) Apc.LaeA and Apc.VeA of the Velvet Complex Govern Secondary Metabolism and Morphological Development in the Echinocandin-Producing Fungus *Aspergillus pachycristatus*. J Ind Microbiol Biotechnol doi:10.1007/s10295-019-02250-x.

Li Y, Chen L, Yue Q, Liu X, An Z, Bills GF (2015) Genetic Manipulation of the Pneumocandin Biosynthetic Pathway for Generation of Analogues and Evaluation of Their Antifungal Activity. ACS Chem Biol 10(7):1702–1710 doi:10.1021/acschembio.5b00013.

Masurekar PS, Fountoulakis JM, Hallada TC, Sosa MS, Kaplan L (1992) Pneumocandins from *Zalerion arboricola*. II. Modification of product spectrum by mutation and medium manipulation. J Antibiot 45(12):1867–74

Mizuno K, Yagi A, Satoi S, Takada M, Hayashi M (1977) Studies on aculeacin. I. Isolation and characterization of aculeacin A. J Antibiot 30(4):297–302

Morris S, Schwartz R, Sesin D, Masurekar P, Hallada T, Schmatz D, Bartizal K, Hensens O, Zink D (1994a) Pneumocandin D_0, a new antifungal agent and potent inhibitor of *Pneumocystis carinii*. J Antibiot 47(7):755–764

Morris SA, Schwartz RE, Sesin DF, Masurekar P, Hallada TC, Schmatz DM, Bartizal K, Hensens OD, Zink DL (1994b) Pneumocandin D_0, a New Antifungal Agent and Potent Inhibitor of *Pneumocystis carinii*. J Antibiot 47(7):755–64 doi:10.7164/antibiotics.47.755.

Mukhopadhyay T, Ganguli BN, Fehlhaber HW, Kogler H, Vertesy L (1987) Mulundocandin, a new lipopeptide antibiotic. II. Structure elucidation. J Antibiot 40(3):281–9

Noble HM, Langley D, Sidebottom PJ, Lane SJ, Fisher PJ (1991) An echinocandin from an endophytic *Cryptosporiopsis* sp. and *Pezicula* sp. in *Pinus sylvestris* and *Fagus sylvatica*. Mycol Res 95(12):1439–1440 doi:http://dx.doi.org/10.1016/S0953-7562(09)80401-2.

Nordberg H, Cantor M, Dusheyko S, Hua S, Poliakov A, Shabalov I, Smirnova T, Grigoriev IV, Dubchak I (2014) The genome portal of the Department of Energy Joint Genome Institute: 2014 updates. Nucleic Acids Res 42(Database issue):D26–D31 doi:10.1093/nar/gkt1069.

Otani M, Satoi S, Takaka M (1978) Antibiotic Aculeacin – A.alpha,-A.gamma,-D.alpha and -D.gamma and their Production. Canada Patent CA 1110561, 1981-10-13.

Peláez F, Collado J, Platas G, Overy DP, Martín J, Vicente F, González del Val A, Basilio A, De la Cruz M, Tormo JR, Fillola A, Arenal F, Villareal M, Rubio V, Baral HO, Galán R, Bills GF (2011) Phylogeny and intercontinental distribution of the pneumocandin-producing anamorphic fungus *Glarea lozoyensis*. Mycology 2(1):1–17 doi:10.1080/21501203.2010.544334.

Roy K, Mukhopadhyay T, Reddy GC, Desikan KR, Ganguli BN (1987) Mulundocandin, a new lipopeptide antibiotic. I. Taxonomy, fermentation, isolation and characterization. J Antibiot 40(3):275–80

Satoi S, Yagi A, Asano K, Mizuno K, Watanabe T (1977) Studies on aculeacin. II. Isolation and characterization of aculeacins B, C, D, E, F and G. J Antibiot 30(4):303–7

Schwartz RE, Giacobbe RA, Bland JA, Monaghan RL (1989) L-671,329, a New Antifungal Agent. I. Fermentation and Isolation. J Antibiot 42(2):163–167

Schwartz RE, Sesin DF, Joshua H, Wilson KE, Kempf AJ, Goklen KA, Kuehner D, Gailliot P, Gleason C, White R, Inamine E, Bills G, Salmon P, Zitano L (1992) Pneumocandins from *Zalerion arboricola*. I. Discovery and isolation. J Antibiot 45(12):1853–66

Strobel GA, Miller RV, Martinez-Miller C, Condron MM, Teplow DB, Hess WM (1999) Cryptocandin, a potent antimycotic from the endophytic fungus *Cryptosporiopsis* cf. *quercina*. Microbiology 145(8):1919–1926 doi:10.1099/13500872-145-8-1919.

Traber R, Keller-Juslén C, Loosli H-R, Kuhn M, Von Wartburg A (1979) Cyclopeptid-Antibiotika aus *Aspergillus*-Arten. Struktur der Echinocandine C und D. Helv Chim Acta 62(4):1252–1267 doi:10.1002/hlca.19790620436.

Untereiner WA, Yue Q, Chen L, Li Y, Bills GF, Stepanek V, Reblova M (2019) *Phialophora* Section *Catenulatae* Disassembled: New Genera, Species, and Combinations and a New Family

Encompassing Taxa with Cleistothecial Ascomata and Phialidic Asexual States. Mycologia:1–30 doi:10.1080/00275514.2019.1663106.
Wingfield BD, Bills GF, Dong Y, Huang W, Nel WJ, Swalarsk-Parry BS, Vaghefi N, Wilken PM, An Z, de Beer ZW, De Vos L, Chen L, Duong TA, Gao Y, Hammerbacher A, Kikkert JR, Li Y, Li H, Li K, Li Q, Liu X, Ma X, Naidoo K, Pethybridge SJ, Sun J, Steenkamp ET, van der Nest MA, van Wyk S, Wingfield MJ, Xiong C, Yue Q, Zhang X (2018) Draft Genome Sequence of *Annulohypoxylon stygium*, *Aspergillus mulundensis*, *Berkeleyomyces basicola* (syn. *Thielaviopsis basicola*), *Ceratocystis smalleyi*, Two *Cercospora beticola* Strains, *Coleophoma cylindrospora*, *Fusarium fracticaudum*, *Phialophora* cf. *hyalina*, and *Morchella septimelata*. IMA Fungus 9(1):199–223 doi:10.5598/imafungus.2018.09.01.13.
Xu J, Chen Y, Ji X, Gao X, Liu S, Zhang Z (2012) High yield antibiotics producing fungus strain, preparation method and use thereof. United States Patent US 20120258498,
Yue Q, Chen L, Zhang X, Li K, Sun J, Liu X, An Z, Bills GF (2015) Evolution of Chemical Diversity in Echinocandin Lipopeptide Antifungal Metabolites. Eukaryot Cell 14(7):698–718 doi:10.1128/EC.00076-15.
Yue Q, Li Y, Chen L, Zhang X, Liu X, An Z, Bills GF (2018) Genomics-driven discovery of a novel self-resistance mechanism in the echinocandin-producing fungus Pezicula radicicola. Environ Microbiol 20(9):3154–3167 doi:10.1111/1462-2920.14089.
Zou SP, Zhong W, Xia CJ, Gu YN, Niu K, Zheng YG, Shen YC (2015) Mutagenesis breeding of high echinocandin B producing strain and further titer improvement with culture medium optimization. Bioprocess Biosyst Eng 38(10):1845–54 doi:10.1007/s00449-015-1425-4.
